# Screening for Allergy

**Published:** 1988-02

**Authors:** E. C. Hamlyn

**Affiliations:** Ivy Bridge Devon PL21 0DQ


### Correspondence

**Screening for Allergy**

Dear Sir,

Dr. Burton has asked how to screen a patient for allergy in the absence of a reliable test.

The steps for allergy screening are as follows:

1. Search for a family history of allergy. When there are allergies on both sides of the family an allergic diathesis is very likely.
2. As a new born baby was the patient easy to rear or did it cry excessively, have colic, bring back its feed or show signs of being "difficult"?
3. Did the patient as a child have its tonsils or adenoids removed, were grommets needed? In childhood did the patient have asthma or eczema, recurrent infections, frequent colds, convulsions, tantrums, tummy aches, growing pains, nightmares, bed wetting or any other indication of allergy?
4. Has the patient ever suffered from migraine, catarrh, sinusitis, hay fever, urticaria, mouth ulcers, an irritable bowel or trouble with bright lights?
5. does the patient now have a low energy level, wake tired in the morning, feel sleepy in the day, have problems with sleep such as snoring, and are there any addictions?
6. Where there is no clinical evidence of an allergic diathesis find out what happened immediately prior to the onset of the illness. Look for a poisoning, a viral illness or any incident which could have damaged the immune system and so set the scene for an allergic illness.
7. Where there is a strong suspicion that the patient does have an allergic diathesis then the final test is to put the patient on an elimination diet for two weeks.
8. If the patient then experiences initial withdrawal symptoms followed by amelioration of his original symptoms the screening for allergy is positive.

Yours faithfully,

Dr. E. C. Hamlyn.
Ivy Bridge
Devon
PL21 0DQ

